# Factors Associated with Malignant Gastric Ulcers: A 10-year Retrospective Cohort Study

**DOI:** 10.2147/IJGM.S593454

**Published:** 2026-05-29

**Authors:** Anas Zayad, Yousef Yahia, Prem Chandra, Ibrahim Obeidat, Mohamad Safieh, Sadi Alnakhala, Husam A Saffo

**Affiliations:** 1 Internal Medicine Department, Hamad Medical Corporation, Doha, Qatar; 2 Gastroenterology and Hepatology Department, Hamad Medical Corporation, Doha, Qatar; 3 Medical Research Center, Hamad Medical Corporation, Doha, Qatar

**Keywords:** gastric ulcer, gastric cancer, malignancy risk, ulcer size, endoscopy, *Helicobacter pylori*, real-world study, retrospective cohort, predictive factors, middle east epidemiology

## Abstract

**Background:**

Gastric ulcers carry a recognized risk of underlying malignancy. Regional evidence remains limited in the Middle East, where historically high *Helicobacter pylori* prevalence contrasts with comparatively low gastric cancer incidence. This study evaluated the prevalence of malignant gastric ulcers and identified clinical and endoscopic predictors of malignancy.

**Methods:**

A retrospective cohort of all patients diagnosed with gastric ulcer between 2014 and 2024 at Hamad Medical Corporation was analyzed. Local protocols mandate biopsy and follow-up endoscopy, although real-world adherence varies. Collected variables included demographics, ulcer morphology, *H. pylori* status, and clinical exposures. Ulcer size was categorized as small (<1.25 cm), medium (1.25–3.0 cm), and large (>3.0 cm). Logistic regression and ROC analyses were used to evaluate predictors and model performance.

**Results:**

Among 473 patients, 39 (8.2%) had malignant ulcers. Ulcer size was the strongest predictor of malignancy; medium and large ulcers demonstrated markedly increased odds in univariate and multivariable analyses. Age, sex, smoking, alcohol use, *H. pylori* status, and ulcer location showed no independent associations. The multivariable model achieved an AUC of 0.868, compared with 0.790 using ulcer size alone. Adenocarcinoma was the predominant subtype.

**Conclusion:**

Malignant gastric ulcers accounted for a meaningful minority of ulcer presentations. Ulcer size was the dominant predictor of malignancy, while demographic, lifestyle, and microbial factors added limited discriminatory value. These findings support emphasizing ulcer size in risk stratification and ensuring universal biopsy of all gastric ulcers, with timely follow-up for medium and large lesions.

## Introduction

Gastric cancer (GC) remains a major global health concern, ranking as the fifth most common malignancy and the fourth leading cause of cancer-related death worldwide.^1^,^2^ Peptic ulcer disease (PUD) is defined as a break in the mucosal integrity of the stomach or duodenum that extends through the muscularis mucosae.^3^ Although global prevalence has declined, PUD continues to be clinical relevant because of complications such as bleeding, perforation, and gastric outlet obstruction. Gastric ulcers, in particular, warrant careful evaluation because of their established association with underlying malignancy.^3^,^4^

The pathogenesis of gastric ulcer is multifactorial. *H. pylori* infection and non-steroidal anti-inflammatory drug use remain the leading etiologic factors worldwide.^5–7^ Chronic NSAID exposure is common among older adults, with up to one-third of long-term users developing ulcer disease.^7^ Smoking further increases ulcer risk, delays healing, and heightens susceptibility to *H. pylori* infection; large epidemiologic studies demonstrate nearly a twofold higher prevalence of PUD among smokers, with a clear dose response relationship. Smoking is also an established risk factor for gastrointestinal malignancies, including gastric carcinoma.^8^,^9^

Although several studies have evaluated factors associated with malignant gastric ulcers, uncertainty remains regarding which clinical and endoscopic features most reliably distinguish malignant from benign lesions. Prior reports have variably identified ulcer size, location, endoscopist suspicion, and endoscopic stigmata such as irregular borders or a dirty base as relevant markers, but these findings have not been fully consistent across cohorts and practice settings. In addition, follow-up strategies remain debated, with differences between guideline recommendations and risk-stratification approaches in the literature. This ongoing variability supports the need for additional population-specific data to clarify which ulcer characteristics are most clinically informative.^10–12^

Early epidemiologic data from Qatar demonstrate historically high *H. pylori* prevalence, with a clinico-histopathologic series reporting 77% positivity among adults undergoing endoscopy.^13^ Across the Middle East, including Qatar, this high infection burden coincides with comparatively low GC incidence, suggesting regional differences in carcinogenic progression. These observations highlight the importance of defining reliable predictors of malignant gastric ulcers in local populations, particularly given the limited regional data compared with Western and East Asian cohorts.

Across the Middle East and North Africa (MENA) region, *Helicobacter pylori* infection remains highly prevalent, with reported adult prevalence rates reaching up to 36.8–94%, largely driven by socioeconomic and environmental factors.^14^ Despite this high burden, gastric cancer incidence in the region remains comparatively low and heterogeneous, ranging from 3.4 per 100,000 in some countries to higher rates in others, suggesting regional differences in carcinogenic progression.^15^ Similar observations have been reported in Qatar, where studies demonstrate a substantial prevalence of *H. pylori* infection among dyspeptic patients, supporting the need to better characterize local risk factors associated with malignant transformation.^16^

Ulcer size has been proposed as a clinically relevant parameter in differentiating benign from malignant gastric ulcers, with larger lesions more likely to harbor malignancy due to increased tumor burden, infiltrative growth patterns, and delayed healing. Several endoscopic studies have suggested that ulcer size may correlate with malignant potential and should be considered alongside other clinical and endoscopic features during evaluation.^17^,^18^

Most studies examining malignant gastric ulcers originate from high incidence regions, while evidence from Arab populations remains scarce. This study examined all patients diagnosed with gastric ulcer at a national referral center in Qatar over ten years. The primary objective was to determine the prevalence of malignant gastric ulcers, and the secondary objective was to identify clinical and endoscopic predictors of malignancy.

## Methods

### Study Design and Setting

This retrospective study was conducted over a 10-year period, from January 2014 to January 2024, including all patients diagnosed with gastric ulcer at Hamad Medical Corporation (HMC), Qatar. Institutional protocols mandate biopsy of endoscopically detected gastric ulcers; however, in a minority of cases, biopsy was not obtained due to clinical judgment, procedural factors, or loss to follow-up. The HMC recommends repeat endoscopic evaluation within 6–8 weeks for all gastric ulcers to confirm healing and re-biopsy any persistent lesions. These pathways aim to provide guideline-concordant management of gastric ulcers across Qatar’s public healthcare system.^19^,^20^

### Data Collection

Data were extracted from electronic medical records (EMR) using a standardized data collection template. The variables collected included demographic information (age and sex), ulcer characteristics (location, size, and number), endoscopic findings, histopathology results, *H. pylori* status, NSAID and aspirin use, smoking, and alcohol intake, and outcomes (healing status and presence of malignant histology). Ulcer size was categorized according to pre-established endoscopic thresholds aligned with the Edinburgh Gastric Ulcer Score (EGUS) diagnostic framework.^11^ Lesions were measured during endoscopy based on the largest diameter, estimated using open biopsy forceps as a reference standard, as routinely applied in clinical practice, ulcers and classified into three groups: small ulcers (<1.25 cm), medium ulcers (1.25–3.0 cm), and large ulcers (>3.0 cm). Age was analyzed both as a continuous variable and as a binary variable using the EGUS validated threshold of ≥68 years, based on the original EGUS derivation study, which identified 68 years as the optimal discriminatory cutoff for malignant gastric ulceration. Patients with a prior or known diagnosis of gastric malignancy at the time of index endoscopy were excluded to ensure assessment of incident malignant ulcers. Patients were followed through outpatient clinic visits and electronic medical records, including repeat endoscopy findings and histopathology results when available. Biopsy was routinely performed for all gastric ulcers as per institutional practice. However, it was not obtained in a minority of cases due to clinical judgment, procedural factors, or loss to follow-up.

### Statistical Analysis

Continuous variables were summarized as means with standard deviations (SD) or medians with interquartile ranges (IQR), depending on data distribution. Categorical variables were expressed as frequencies and percentages. Comparisons between benign and malignant ulcers used Pearson Chi-square, Yates’s Chi-squared or Fisher’s exact tests for categorical variables and Student’s *t* test or Mann Whitney *U*-test for continuous variables, as appropriate.

Univariate logistic regression analyses were performed to identify potential predictors associated with malignant gastric ulcers. Ulcer size was forced into all multivariable models. Age ≥68 years, defined a priori from the EGUS threshold, and ulcer location were also included regardless of univariate p values, while other variables with p<0.10 in univariate analysis or if they were determined to be clinically meaningful were considered for inclusion. Duodenal ulcers were excluded due to complete separation, since no malignant cases occurred. The goodness-of-fit of the developed logistic regression models was estimated using the Hosmer and Lemeshow test (good fit if p>0.05) and the Omnibus test (p≤0.05). The explained variance of the model was estimated using Cox and Snell’s R2 and Nagelkerke’s R2. Furthermore, the predictive ability of the developed logistic regression models was estimated using the area under the curve (AUC) derived via receiver operating characteristic (ROC) analysis. All analyses were conducted using SPSS version 29.0 (IBM Corp., Armonk, NY) and STATA version 16, and a p value <0.05 was considered statistically significant.

### Ethical Considerations

The study was conducted in accordance with the Declaration of Helsinki and was approved by the Institutional Review Board (IRB) and the Medical Research Center of Hamad Medical Corporation (MRC-01-25-609). The requirement for informed consent was waived due to the retrospective nature of the study and the use of anonymized data.

## Results

### Patient Characteristics

A total of 473 patients diagnosed with gastric ulcer were included, of whom 434 (91.8%) had benign histology and 39 (8.2%) had malignant histology. The mean age of the cohort was 52.3 ± 14.2 years. The proportion of patients aged ≥68 years did not differ between malignant and benign groups, indicating limited discriminatory value for this threshold. Males constituted 68.7% of the cohort, and ethnic distribution was similar across groups.

Most ulcers were small in size (<1.25 cm), comprising 83.9% of all lesions. Large ulcers (≥3 cm) were markedly more common among malignant cases compared with benign cases (41.0% vs 4.4%, p < 0.001). Ulcer location also differed between groups, with non-antral sites more frequently associated with malignancy (59.0% vs 28.8%, p < 0.001). Most patients (68.8%) presented with a single ulcer, and the number of ulcers did not differ between benign and malignant cases (p = 0.254).

Gastrointestinal bleeding was the most common indication for endoscopy (38.5%), followed by dyspepsia (24.9%) and anemia (9.3%). Dyspepsia was more frequently reported in malignant cases (48.7%) than benign cases (22.8%). *Helicobacter pylori* infection was detected in 33.6% of the cohort, with no significant difference between malignant and benign ulcers (20.5% vs 34.8%, p = 0.071). Smoking, alcohol use, and NSAID or aspirin exposure were not significantly associated with risk of developing GC. The denominators for these variables varied due to incomplete documentation in the electronic medical records.

Biopsy was performed in 89% of patients. All malignant ulcers were biopsied, compared with 88% of benign ulcers. Histopathology from the index endoscopy identified 32 malignant cases (7.6%), and an additional seven malignancies were detected on follow-up endoscopy, yielding a total of 39 malignant ulcers. These seven cases had initially non-malignant histopathology and therefore represent false-negative index evaluations or missed diagnoses due to sampling error and lesion heterogeneity. Additionally, factors such as incomplete adherence to follow-up protocols, and loss to follow-up may have contributed to delayed diagnosis. Benign ulcers demonstrated a wide spectrum of non-neoplastic patterns, most commonly non–*H. pylori* gastritis (46.2%), *H. pylori* gastritis (35.2%), and chemical gastropathy (11.3%). Intestinal metaplasia, dysplasia, and atypia were rare and occurred almost exclusively in benign ulcers. Among malignant ulcers, adenocarcinoma was the predominant subtype (59.0%), followed by lymphoma (35.9%) and a small number of other malignancies (5.1%). Endoscopic treatment was performed in 7.8% of patients, all within the benign ulcer group; no malignant ulcer required endoscopic therapy. All duodenal ulcers (n = 57) occurred exclusively in benign cases, resulting in complete separation and exclusion of this variable from regression analysis. Baseline patient characteristics are summarized in Table 1.

**Table 1:** Baseline Characteristics of the Study Population (n = 473) *

Predictor Variable	Total (n=473)	Benign (n=434)	Malignant (n=39)	P-value
Age, mean (SD)	52.3 (14.2)	52.36 (14.21)	51.76 (15.18)	0.804
Male, n (%)	325 (68.7)	297 (68.4)	28 (71.8)	0.665
Ethnicity, n (%)				0.512
Arab	294 (62.2)	271 (62.4)	23 (59.0)	
Asian	152 (32.1)	137 (31.6)	15 (38.5)	
Others	27 (5.7)	26 (6.0)	1 (2.6)	
Ulcer Size (cm), n (%)				<0.0001
<1.25	397 (83.9)	386 (88.9)	11 (28.2)	
1.25–2.99	41 (8.7)	29 (6.7)	12 (30.8)	
>3.00	35 (7.4)	19 (4.4)	16 (41.0)	
Non-antral ulcer location, n (%)	148 (31.3)	125 (28.8)	23 (59.0)	<0.0001
Number of ulcers, n (%)	n=464	n=425	n=39	0.254
1	319 (68.8)	286 (67.3)	33 (84.6)	
2	63 (13.6)	59 (13.9)	4 (10.3)	
3	56 (12.1)	55 (12.9)	1 (2.6)	
More than 3	26 (5.5)	25 (5.8)	1 (2.6)	
Reason for Endoscope n (%)				0.010
GI bleed	182 (38.5)	173 (39.9)	9 (23.1)	
Dyspepsia	118 (24.9)	99 (22.8)	19 (48.7)	
Anaemia	44 (9.3)	39 (9.0)	5 (12.8)	
Weight loss	11 (2.3)	10 (2.3)	1 (2.6)	
Others	118 (25)	113 (26.1)	5 (12.9)	
Smoking History, n (%)	84/432 (19.4)	75/395 (19.0)	9/37 (24.3)	0.433
Alcohol intake History, n (%)	30/385 (7.8)	27/352 (7.7)	3/33 (9.1)	0.771
Family History of gastric cancer, n (%)	15/310 (4.8)	13/277 (4.7)	2/33 (6.1)	0.729
H.pylori infection, n (%)	158/470 (33.6)	150/431 (34.8)	8/39 (20.5)	0.071
NSAID/aspirin status at time of EGD, n (%)	n=471	n=432	n=39	0.089
No	305 (64.8)	273 (63.2)	32 (82.1)	
NSAID	69 (14.6)	67 (15.5)	2 (5.1)	
Aspirin	81 (17.2)	76 (17.6)	5 (12.8)	
Both Aspirin and NSAID	16 (3.4)	16 (3.7)	0 (0.0)	
Endoscopic treatment, n (%)	37 (7.8)	37 (8.5)	0 (0.0)	0.058
Duodenal Ulcer,n (%)	57 (12.1)	57 (13.1)	0 (0.0)	0.016
Ulcer Biopsy Taken, n (%)	421 (89.0)	382 (88.0)	39 (100.0)	0.022
Histopathology findings in EGD, n(%)	n=420	n=381	n=39	<0.001
Normal	21 (5.0)	21 (5.5)	0 (0.0)	
Chemical gastropathy	44 (10.5)	43 (11.3)	1 (2.6)	
*H. pylori* gastritis	135 (32.1)	134 (35.2)	1 (2.6)	
Cancer	32 (7.6)	0 (0.0)	32 (82.1)	
Non-*H.pylori* gastritis	177 (42.1)	176 (46.2)	1 (2.6)	
Intestinal metaplasia	7 (1.7)	6 (1.6)	1 (2.6)	
Dysplasia	1 (0.2)	0 (0.0)	1 (2.6)	
Healing	2 (0.5)	1 (0.3)	1 (2.6)	
Atypia	1 (0.2)	0 (0.0)	1 (2.6)	
Type of Cancer, n (%)				
Adenocarcinoma			23 (59.0%)	
Lymphoma			14 (35.9%)	
Others			2 (5.1%)	

Note: *Some variables contained missing values because of incomplete documentation in the medical records.

**Abbreviations**: SD, Standard deviation; GI, Gastrointestinal; *H. pylori*, *Helicobacter pylori*; NSAID, Non-steroidal anti-inflammatory drug; EGD, Esophagogastroduodenoscopy.

### Logistic Regression Analysis of Predictors Associated with Malignant Gastric Ulcers

Univariate logistic regression identified ulcer size as the strongest predictor associated with malignant gastric ulcers as shown in Table 2. Compared with small ulcers, medium-sized ulcers demonstrated markedly increased odds of malignancy (unadjusted OR 14.52, 95% CI 5.90–35.75; p < 0.001), and large ulcers conferred an even higher risk (unadjusted OR 29.55, 95% CI 12.07–72.34; p < 0.001). Ulcer location was also significant in univariate analysis, with non-antral ulcers more likely to be malignant than antral ulcers (unadjusted OR 3.55, 95% CI 1.82–6.95; p =0.003). Age ≥68 years, gender male, smoking, alcohol use, and *H. pylori* infection were insignificantly associated with malignant ulcers in univariate analysis.

**Table 2:** Univariate and Multivariate Logistic Regression for Predictors of Malignancy

Covariate	Comparison (Reference)	Univariate OR (95% CI)	P-value	Multivariate OR (95% CI)	P-value
Age	>68 years versus ≤68 years	1.31 (0.56, 3.10)	0.536	0.56 (0.17, 1.83)	0.335
Sex	Male versus Female	0.85 (0.41, 1.76)	0.665	0.82 (0.31, 2.16)	0.665
Ulcer size	Medium vs small	14.52 (5.90, 35.75)	<0.001	11.67 (3.82, 35.66)	<0.001
Ulcer size	Large vs small	29.55 (12.07, 72.34)	<0.001	33.57 (11.97, 94.19)	<0.001
Ulcer location	Non antral vs antral	3.55 (1.82, 6.95)	0.003	1.41 (0.58, 3.47)	0.451
Smoking	Smoker vs non-smoker	1.37 (0.62, 3.03)	0.434	0.90 (0.27, 2.96)	0.856
Alcohol use	Alcohol use vs no alcohol	1.20 (0.35, 4.20)	0.771	2.17 (0.39, 12.19)	0.377
*H. pylori* infection	Present vs absent	0.49 (0.22, 1.08)	0.076	0.49 (0.17, 1.44)	0.195

**Note**: Duodenal ulcer presence was excluded due to complete separation, with no malignancy cases in this category, preventing valid estimation of odds ratios.

**Abbreviation**: OR, Odds ratio.

In the multivariable logistic regression analysis, ulcer size remained the only independent predictor of malignancy. Medium ulcers (adjusted OR 11.67, 95% CI 3.82–35.66; p < 0.001) and large ulcers (adjusted OR 33.57, 95% CI 11.97–94.19; p < 0.001) both showed strong associations, with large ulcers presenting the greatest risk. The association between non-antral location and malignancy was no longer statistically significant (adjusted OR 1.41, 95% CI 0.58–3.47; p = 0.514). No independent associations were observed for age ≥68 years, gender male, smoking, alcohol use, or *H. pylori* status. Duodenal ulcers were excluded from the analysis because no malignant cases occurred in this subgroup, resulting in complete separation. The multivariate logistic regression model appeared to be well fit (Hosmer–Lemeshow test, P=0.944; and the Omnibus test, p<0.001) and had good predictive validity.

### Receiver Operating Characteristic (ROC) Curves

**Figure 1:**
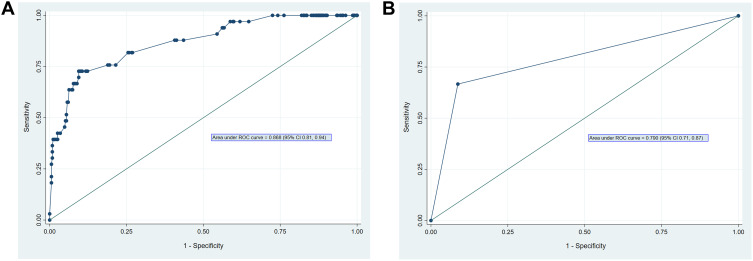
(**A** and **B**) Receiver-operating characteristic (ROC) curves evaluating the performance of ulcer size in predicting malignant gastric ulcers. (**A**) represents the multivariable model including ulcer size and additional covariates, demonstrating strong discriminative ability (AUC 0.868, 95% CI 0.81–0.94). (**B**) represents a simplified model using ulcer size as the sole predictor, showing moderate discriminative ability (AUC 0.790, 95% CI 0.71–0.87). The diagonal line represents the line of no discrimination.

Receiver Operating Characteristic (ROC) analysis was performed to assess the discriminative performance of the predictive models (Figure 1A and B). The full multivariable model, which included ulcer size, ulcer location, age, gender, alcohol use, smoking, and *H. pylori* status, demonstrated excellent discriminative ability and performance in predicting ulcer malignancy with an area under the curve (AUC) of 0.868 (95% CI 0.81–0.94) (Figure 1A).

To evaluate the individual contribution of ulcer size, a second ROC curve was generated using a model with ulcer size as the sole predictor. This simplified model produced an AUC of 0.790 (95% CI 0.71–0.87), indicating that ulcer size alone provides strong discriminatory power, although performance was enhanced when additional covariates were included.

## Discussion

In this 10-year cohort, malignant gastric ulcers were identified in 8.2% of patients, representing a meaningful cancer burden within a low-incidence regional setting. Ulcer size was the strongest predictor of malignancy, with medium- and large-sized ulcers showing markedly increased risk in multivariable analysis. In contrast, demographic variables, lifestyle factors, NSAID use, ulcer location, and *Helicobacter pylori* status did not demonstrate independent associations. ROC analyses supported these findings, showing that ulcer size provided the greatest discriminatory ability, with other variables offering only modest incremental value. Together, these results establish ulcer size as the primary determinant of malignancy risk, underscoring its central role in risk stratification, surveillance planning, and timely oncologic referral.

Ulcer size has long been recognized as a key malignant feature, consistent with established tools such as the Edinburgh Gastric Ulcer Score (EGUS), which incorporates size for its high reproducibility and diagnostic yield.^11^ Larger ulcers likely reflect deeper tissue involvement or ulceration occurring over infiltrative neoplasia, explaining the persistence of size as a significant predictor even after adjusting for other factors. Although EGUS identifies age ≥68 years as a risk factor, this association was not seen in our dataset. This likely reflects the limited number of malignant cases and the younger age profile of the Qatar population, reducing the proportion of patients who meet the EGUS high-risk age threshold.

Ulcer location showed significance in univariate analysis, with non-antral ulcers more common among malignant cases. However, this association disappeared after adjustment, indicating that ulcer size accounted for most of the observed risk. Traditional clinical variables, including smoking, alcohol use, and *H. pylori* status, also showed no independent associations, although incomplete testing and limited power must be acknowledged.

The lack of an independent association between *H. pylori* and malignant ulcers in our cohort is consistent with regional observations, where high infection prevalence coincides with comparatively low gastric cancer incidence.^15^ Qatar follows this pattern, with low national gastric cancer rates despite historically high *H. pylori* prevalence on endoscopic testing.^13^ Similar findings have been reported in other Middle Eastern populations, including an Omani study showing no significant association with cancer risk.^21^ Several factors may contribute to this attenuated carcinogenic progression, including variation in bacterial virulence profiles,^22^,^23^ host genetic susceptibility,^24^,^25^ and regional environmental exposures. However, these mechanisms cannot be confirmed in this study because bacterial genotypes, host polymorphisms, and standardized *H. pylori* testing were not assessed. Taken together, these considerations likely explain the limited discriminatory value of *H. pylori* status in this cohort.

Our findings regarding ulcer location align with regional and international evidence showing that proximal gastric ulcers, particularly those in the corpus, fundus, or cardia, carry higher malignant potential. This pattern is supported by a large UK cohort in which non-antral location remained an independent risk factor and by classic site-specific studies reporting higher malignant rates in cardia and corpus ulcers.^10^,^11^ Contemporary data also identify proximal location as a high-risk endoscopic feature, particularly in the presence of larger or morphologically atypical ulcers.^26^ In our multivariable analysis, however, ulcer location lost significance, suggesting that ulcer size captured most of the risk. Clinically, these findings reinforce ulcer size as the principal feature warranting prioritization during evaluation.

This study has several limitations. These include its retrospective design, missing data for some clinical variables, non-standardized *H. pylori* testing, imperfect adherence to recommended biopsy and follow-up protocols, and the relatively small number of malignant cases, which restricts statistical power. Furthermore, this is a single-center study, which may limit generalizability. Larger, prospective, multicenter studies with uniform diagnostic and sampling methods are needed to refine predictors of malignancy among gastric ulcer patients in this region.

## Conclusion

Malignant gastric ulcers represented a meaningful minority of gastric ulcer presentations in this cohort. Ulcer size was the strongest and most consistent predictor of malignancy. These findings support emphasizing ulcer size in endoscopic risk stratification and ensuring universal biopsy of all gastric ulcers, with timely follow-up for medium and large lesions. The retrospective single-center design should be considered when interpreting these results.

## Data Availability

Data can be obtained from the corresponding author upon request.
